# Neurobehavioral changes in mice offspring exposed to green tea during fetal and early postnatal development

**DOI:** 10.1186/s12993-017-0128-1

**Published:** 2017-06-01

**Authors:** Jamaan Ajarem, Gawaher Al Rashedi, Mohamed Mohany, Ahmed Allam

**Affiliations:** 1 Department of Zoology, College of Sciences, Faculty of Science, King Saud University, P.O. Box 2455, Riyadh, 11451 Saudi Arabia; 2 Department of Biology, College of Sciences, Hail University, Hail, Saudi Arabia; 3 Department of Zoology, College of Sciences, Faculty of Science, Beni-Suef University, Beni-Suef, 65211 Egypt

**Keywords:** Green tea, Sensory motor reflexes, Offspring, Anxiety, Mice

## Abstract

**Background:**

Green tea extract (GTE) has various health promoting effects on animals and humans. However, the effects of perinatal exposure to GTE on the behavioral aspects of offspring have not been elucidated thus far. GTE was provided for pregnant female mice at concentrations of either 20 or 50 g/L, beginning the day of conception until the third week after delivery, postnatal day 22 (PD 22). Mice pups were subjected to behavioral testing to assess sensory motor reflexes, locomotion, anxiety, and learning on various postnatal days.

**Results:**

Perinatal exposure to GTE resulted in a significant reduction in body weight, as well as earlier body hair appearance and opening of the eyes. Sensory motor reflexes exhibited faster responses and significant stimulatory effects in pups exposed to GTE. During the adolescent period, male and female offspring exhibited increased locomotor activity (on PD 22), reduced anxiety and fear (on PD 25), and enhanced memory and learning abilities (on PD 30), all in both GTE treated groups. All blood counts (RBCs, WBCs, Hb, and platelets), and glucose, cholesterol, triglyceride, and low density lipoprotein concentrations were significantly lower in the GTE-treated pups; however, there was no effect on high density lipoprotein levels.

**Conclusion:**

Our data provide evidence that the high dose of GTE (50 g/L) had higher anxiolytic properties and positive effects on locomotor activities and sensory motor reflexes, as well as learning and memory of the offspring than the low dose of GTE (20 g/L).

## Background

Green tea (GT) is a beverage that is widely consumed worldwide. It can be prepared from the dried leaves of the plant *Camellia sinensis* [1]. People in some countries in Asia and the Middle East prefer to drink green tea. Numerous research studies have elucidated its benefits [2], and over the last few decades, substantial attention has been paid to GT drinkers in these countries because of its considerable health significance. GT leaves are rich in different polyphenols, including flavonols and flavonoids [3]. In addition, GT contains a group of catechins, including epicatechin, epicatechin-3-gallate, epigallocatechin, and epigallocatechin-3-gallate (EGCG). A substantial number of studies have demonstrated the various health benefits of GT, such as reduction of cancer risk [4], treatment of obesity [5], treatment of diabetes [6], inhibition of inflammation [7], increase levels of antioxidants [8], cholesterol lowering [9] and prevention of degenerative brain changes [10]. Most of these health-promoting effects are primarily based on the antioxidant properties of GT polyphenols and other active components.

In this context, a recent study revealed that supplementation of GTE (GTE) during lactation suppressed macrophage infiltration and restored insulin secretion in rat offspring exposed to maternal protein restriction [2]. Drinking GT during pregnancy is not recommended because of the caffeine content and the little research available on the effects on human fetuses. However, it has been reported that GT catechin supplementation during pregnancy caused no fetal malformations or variation in development [1].

Previous studies have shown that GT supplementation ameliorated neurobehavioral aberrations in valproate-induced autism in animals [11], alleviated cerebral ischemia/reperfusion injury [12], and increased locomotor activity in mice [13]. Moreover, GT catechins have been reported to improve spatial cognition learning ability in rats [14]. GT polyphenols improved the behavior of Alzheimer’s disease-like symptoms in mice induced by d-galactose and Abeta25-35 [15]. In particular, behavioral studies in mice indicated that EGCG, which is a main component of GT had anxiolytic activity [16]. Recent investigations also showed the antidepressive-like activity of green tea in adult mice for instance against post-stroke depression [17]. Most of these studies were conducted on adult animals and very few studies have explored the impact of GT during pregnancy or lactation. Therefore, the goal of this study was to determine the effect of perinatal exposure of GTE on morphological stages of growth, behavioral alterations, and changes in blood biochemistry of the resulting offspring.

## Methods

### Experimental animals

Male and virgin female Swiss–Webster strain mice (8–9 weeks old with 25-g body weight) were housed in opaque plastic cages (three females to one male in each cage) measuring 30 × 12 × 11 cm, in the animal facility of the Zoology Department, King Saud University, Riyadh, Saudi Arabia. Animals were kept under a reversed light/dark cycle of 12/12 h. The ambient temperature was regulated between 22 and 25 °C. All animals were allowed to acclimatize for one week prior to the experiment. All animal procedures were performed in accordance with the standards set forth in the guidelines for the care and use of experimental animals by the Committee for the Purpose of Control and Supervision of Experiments on Animals (CPCSEA). The study protocol was approved by the Animal Ethics Committee at King Saud University. Following confirmation of pregnancy (appearance of vaginal plug was considered to be day one of pregnancy), the males were removed from the cages and the females were subjected to experimental treatments. Food and water were available ad libitum, unless otherwise indicated.

### Animal grouping

All pregnant mice were divided into three groups (*n* = 10 per group) as follows: the first group served as the control group and received plain tap water only, and the second and third groups were exposed to 20 and 50 g/L GTE per day, respectively. Each mother ingested 5 mL of the GTE mixture daily: 2.5 mL in the morning and the same amount in the evening by oral intubation. GTE oral administration was begun on day 1 of pregnancy and was continued until postnatal day 22 (PD 22). Thereafter, the mothers were switched to plain tap water.

After delivery, the pups of each experimental group were culled at birth to eight per dam and were left with their mothers until postnatal day 22 (PD 22). During the weaning period, three male and female pups from each litter were color marked to distinguish them from the others, and they were subjected to various behavioral tests (described below) under dim lighting (ca. 8lux). The mother’s water consumption was calculated daily, whereas body weight was recorded every 5 days until postnatal day 21 (PD 21).

### Preparation of GTE

**Fig. 1:**
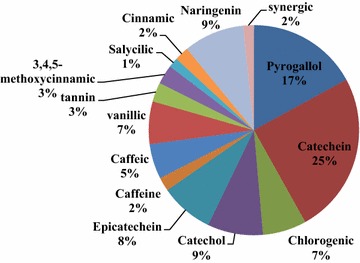
The phenolic compounds content (mg/g) of GTE using liquid chromatography analysis (HPLC) according to Allam et al. [18]

Green tea (GT) was purchased from a local market in Riyadh, Saudi Arabia. The total phenolic compounds in 100 mg of GTE were analyzed using a liquid chromatography “HP1050” and was reported in Fig. 1 as it was published in Allam et al. [18]. GTE was prepared at two dosages (20 and 50 g green tea each in 1000 mL water) [19] by pouring boiling water over green tea leaves and steeping for 15 min. After 1 h, the solution was filtered through Whatman filter paper to remove insoluble material and other impurities, and wrapped in aluminum foil. Fresh GTE was prepared every day.

### Morphological developments in the pups

Physical developmental features, including body weight, opening of the eyes, and appearance of body hair were recorded for the offspring from day 1 after birth (PD 1) through the entire weaning period according to Smart and Dobbing [20].

### Morphological early reflex development in the pups

#### Righting reflex

The righting reflex is a simple and rapid test to assess locomotor abilities in mice. It evaluates general body strength by scoring or measuring the time taken by a neonate to return to their four paws after having been placed in a supine position or on their side. An upper limit of 2 min was set for this test.

#### Rotating reflex

The surface used to measure the rotating reflex was the same as that used for the righting reflex, except it was inclined at an angle of 30°. The pups were placed on this surface with their heads facing down. The time that elapsed until the pup rotated its body through 180° geonegatively and faced its head upward was defined as the rotating time. The upper limit of this test was also set at 2 min.

#### Cliff avoidance reflex

In this test, pups were placed on the edge of a tabletop with the forepaws and face over the edge. The time taken by the pup to back away and turn from the “cliff” was recorded. Again, the upper limit of 2 min was chosen. A latency of 2 min was recorded if the animal fell from the “cliff.”

### Behavioral tests

In this experiment, three types of behavioral tests were assessed as described below.

### Locomotory behaviour

After weaning at PD 22, male and female offspring (*n* = 8) from each group were subjected to ‘Locomotor Activity’ tests in a square-shaped experimental wooden arena measuring 80 × 80 × 30 cm and the floor was divided into 64 squares of equal size. Various observations of behavior, including numbers of squares crossed, wall rears, rears and washes, and duration of locomotion and immobility were observed according to a previous study [21]. The visual observations in the arena lasted 5 min for each animal.

### Fear and anxiety test in plus-maze

Male and female offspring (*n* = 8 per group) were subjected to fear and anxiety tests in a plus-maze at PD 25 of age. The elevated plus-maze (with 2 opened and 2 enclosed arms) is frequently used as a measure for evaluating risk assessment and anxiety behavior of an animal model. In this test, mice were individually placed onto the central platform facing one of the open arms and were observed for 5 min while freely exploring the maze. The animal was considered to have entered an arm when all four limbs were inside the arm. Duration of time spent and number of entries to the center, and open and enclosed arms were measured during the test period.

### Learning and memory test in T-maze

At PD 30 of age, male and female offspring (*n* = 8 per group) were subjected to a learning and memory test in a T-maze. The experimental was conducted according to Maodaa et al. [22]. The maze was a wooden device consisting of three arms, with transparent barriers that could be removed, that formed a T shape: right arm, left arm, and the main long arm. In this test, male and female offspring were starved for 24 h, given only water, prior to the experiment. On the second day, animals were placed in the device for 2 min and food was placed in the left arm. Afterwards, mice were returned to their cages for 3 h and again placed in the T-maze for 5 min per animal. Several parameters of behavior, including latency time to reach the food, time spent in food arm, number of entrances into the food arm, number of entrances into the empty arm, and number of entrances into the main arm, as well as wall rears, rears and washes, and duration of locomotion and immobility were recorder for 5 min for each animal.

### Blood picture and biochemical analysis

For blood profiles and biochemical studies, a subset of developing male and female offspring (*n* = 8 per group) were euthanized at two postnatal ages, PD 15 and PD 30. The animals were euthanized by anesthesia with pentobarbital (60 mg/kg body weight), and two blood samples were immediately collected. The first sample was collected in a heparinized tube (2.25 µL heparin/5 mL blood) for blood profiles. The second sample was collected in a non-heparinized tube and centrifuged for 10 min at 3000 rpm to separate the serum, which was then stored at −80 °C until use in biochemical analyses. For blood profiles, whole samples were analyzed with an automatic Vet abc Animal Blood Counter (Horiba ABX, Montpellier, France) using the hematology kits specified for that instrument (Horiba ABX, France), according to the manufacturer’s instructions. Determination of glucose concentration was conducted according to the method of Siest and Schielef [23], using reagent kits purchased from bioMérieux Chemicals (France), whereas serum triglyceride (TG) concentration was determined according to the method of Fossati and Prencipe [24] using a reagent kit purchased from Reactivos Spinreact Company (Spain). Serum cholesterol concentration was estimated according to the method of Deeg and Ziegenohrm [25] and serum HDL-cholesterol concentration was measured according to the method of Burstein et al. [26] using a reagent kit purchased from Spinreact Company (Spain). Serum LDL-cholesterol concentration was determined according to the method of Friedewald et al. [27].

### Statistical analysis

Data were first tested for normality (using the Anderson–Darling test) and for variance homogeneity prior to any further statistical analyses. For behavioral results, data were presented in tables, expressed as median with ranges, and analyzed using the Mann–Whitney independent test using SPSS software, version 17. Other data (body weight, opening of the eyes, appearance of body hair, and biochemical data) were analyzed using the student *t* test. Data were expressed as the mean ± SEM (standard error of the mean). Differences were considered statistically significant at *p < 0.05, **p < 0.01, and ***p < 0.001.

## Results

### Effects of perinatal exposure to GTE on morphological development of offspring

**Fig. 2:**
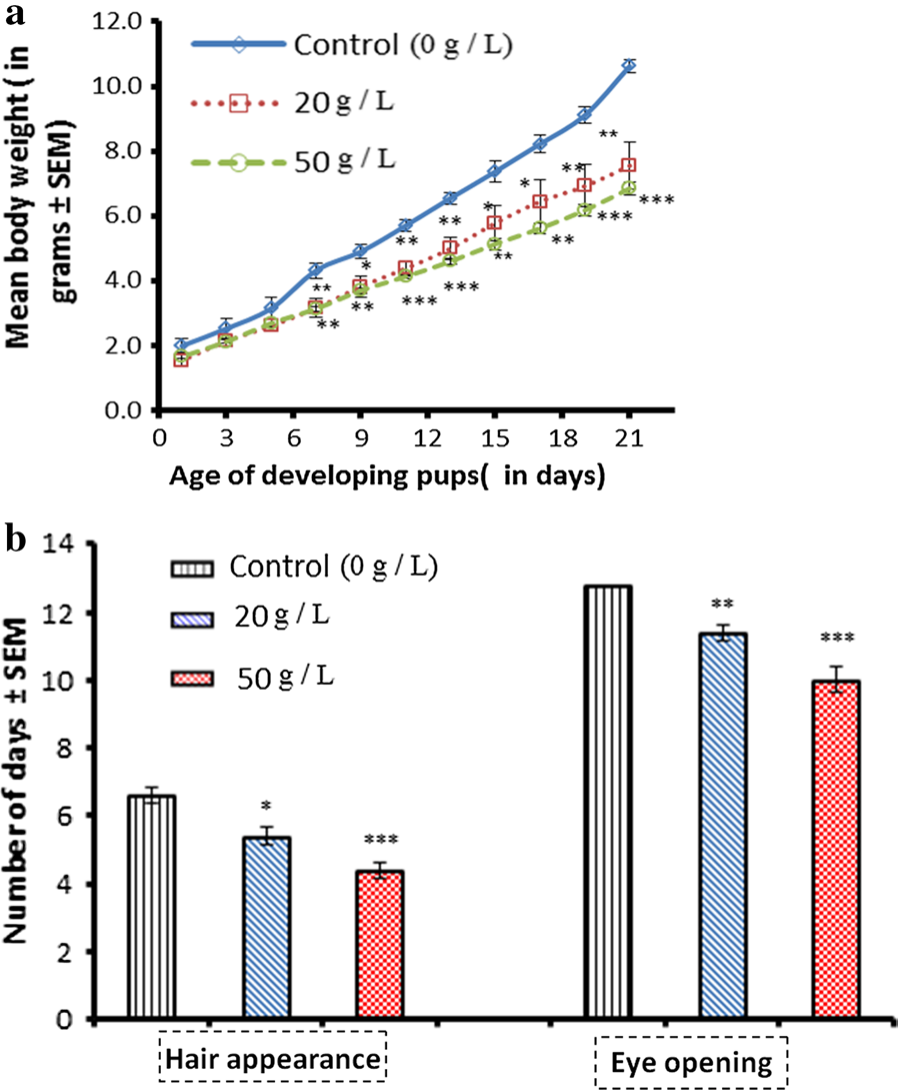
Effects of perinatal GTE (20 and 50 g/L) exposure on body weight (**a**), appearance of hair, and eye opening (**b**) of mice offspring. Data are presented as the mean ± SEM

Physical assessments of body weight, date of first eye opening, and hair appearance were determined in the developing offspring. At birth and until PD 6, no significant differences were noted in the weights of offspring between the control group and treatment groups. From PD 7 to PD 21, body weights were significantly lower in the GTE treatment groups, with minor significant differences in between the two groups exposed to the different GT concentrations (20 and 50 g/L) (Fig. 2a). With regard to other physical landmarks, eye opening and hair appearance were significantly advanced in offspring born to dams exposed to 20 g/L (*p* < 0.001 for both landmarks) and 50 g/L (*p* < 0.05, *p* < 0.01, respectively) of GTE when compared with the control group (Fig. 2b).

### Impacts of perinatal exposure of GTE on neuromotor reflexes of offspring

**Fig. 3:**
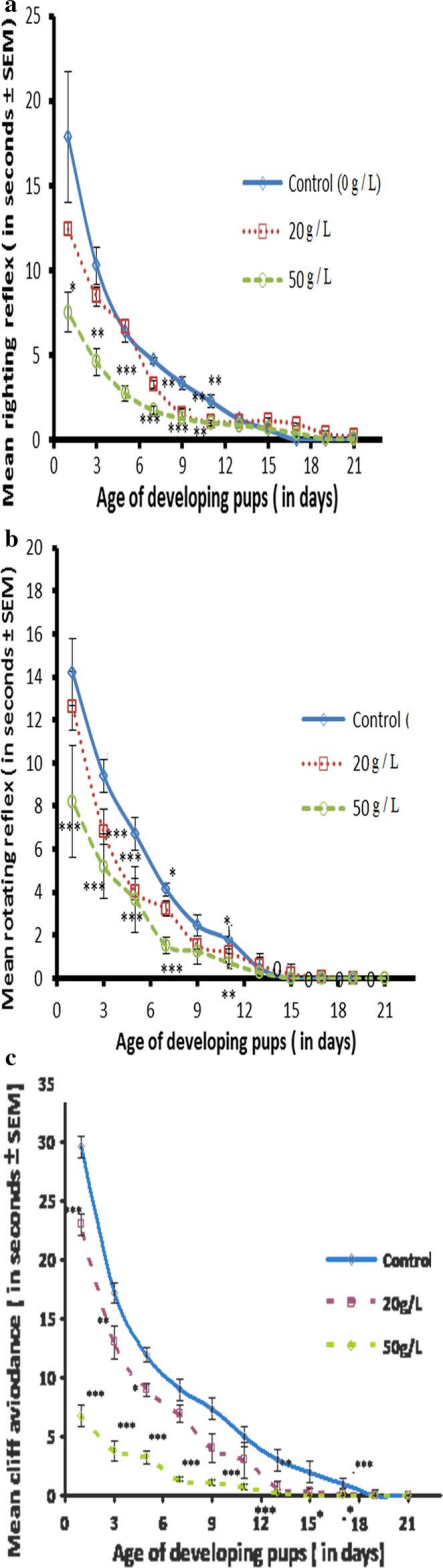
Effects of perinatal GTE exposure on the mean righting reflex (**a**), mean rotating reflex (**b**) and mean cliff avoidance activity (**c**) of mice offspring. Results are expressed as the mean ± SEM. Differences were considered statistically significant at *p < 0.05, **p < 0.01, and ***p < 0.001

The righting, rotating, and cliff avoidance reflexes were determined in the developing offspring to investigate the effect of GTE on the maturation of neuromotor reflexes. We found that exposure to the aqueous extract of GT had a significant stimulatory, dose-dependent effect on righting reflex, rotating reflex, and cliff avoidance activity. The response was greater for offspring born to dams exposed to the higher dose (Fig. 3).

### Effects of perinatal exposure to GTE on locomotor behavior of offspring at PD 22

In male offspring, the locomotor (open-field) activity test was conducted at PD 22 (Table 1). It indicated that perinatal exposure to GTE significantly increased the number of squares crossed, wall rears, and locomotion duration in a dose-dependent manner as compared to that of the controls. The number of body washes (*p* < 0.01) and duration of immobility (*p* < 0.01, p <  0.001 for low- and high-dose groups, respectively) were significantly decreased compared to that of the control, with minor differences between the two dosage groups. With regard to the number of rears in males, there was significant elevation (*p* < 0.01) in the high-concentration (50 g/L) group for GTE, with no significant effect observed for the low-concentration group.

**Table 1:** Effect of perinatal GTE exposure on the locomotor behavior of male mice offspring at postnatal day 22 (*n* = 8 males per group)

Treatment groups	Median number (with ranges) of acts and postures					
	Number of squares crossed	Wall rears	Rears	Wash	Locomotion duration (s)	Immobility duration (s)
Control	186.1 (133–242)	19 (9–33)	4.1 (1–21)	15.6 (8–24)	176.4 (133–266)	123.5 (34–167)
20 g/L	214.8** (188–256)	38** (15–47)	4.9 (2–24)	6.1** (2–19)	231.5** (189–269)	71.5** (31–111)
50 g/L	294.9*** (204–300)	45.1*** (31–68)	10.4** (3–26)	5.1** (3–13)	275.1*** (194–288)	35*** (12–106)

Statistical comparisons were conducted using the Mann–Whitney U test

** and *** indicate statistically significant differences at *p* < 0.01 and *p* < 0.001, respectively

The locomotor activity test for female mice (Table 2) showed a significant elevation in the number of squares crossed, wall rears, rears, and locomotion duration in a dose-dependent manner when compared with that of the control group. However, immobility duration (*p* <  0.05, *p* <  0.001 for low- and high-dose groups, respectively) was significantly decreased, with minor differences between the two concentration groups. The number of body washes for females significantly decreased (*p* <  0.05) in the low dose (20 g/L), but no change was observed in the high-dose group (50 g/L).

**Table 2:** Locomotory behavior of female mice offspring at postnatal day 22 after perinatal exposure to aqueous extract of green tea (*n* = 8 females per group)

Treatment groups	Median number (with ranges) of acts and postures					
	Number of squares crossed	Wall rears	Rears	Wash	Locomotion duration (s)	Immobility duration (s)
Control	168.5 (111–233)	15.3 (4–26)	2 (1–5)	10 (2–19)	169.2 (126–229)	130.8 (71–174)
20 g/L	204.5* (172–245)	29.5* (29–41)	4.5* (2–8)	6.4* (2–16)	223* (164–267)	76.9* (33–136)
50 g/L	268.5** (184– 277)	40.5*** (27–61)	12.3*** (4–19)	10 (2–19)	244*** (198–274)	56.5*** (26–102)

Statistical comparisons were conducted using the Mann–Whitney U test

*, ** and *** indicate statistically significant differences at *p* < 0.05, *p* < 0.01 and *p* < 0.001, respectively

### Anxiety behavior in the elevated plus-maze test in offspring exposed to GTE

**Fig. 4:**
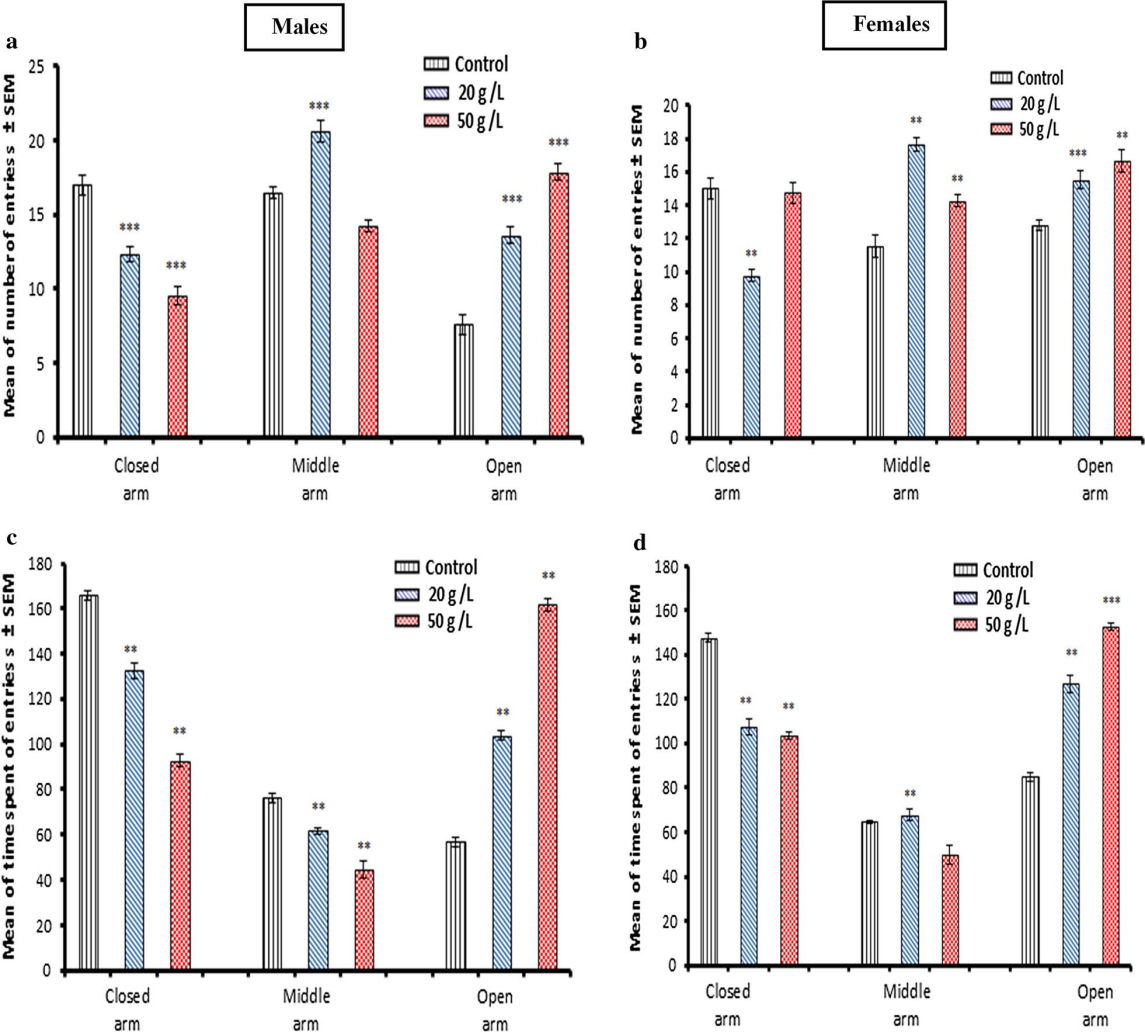
Anxiety-like behavior in the elevated plus-maze. The number of entries (**a**, **b**) and time spent (**c**, **d**) in the arms for mice offspring at postnatal day 25 following perinatal exposure to aqueous extract of green tea. Data are presented as the mean ± SEM (*n* = 8). ** and *** indicate statistically significant differences at *p* < 0.01 and *p* < 0.001, respectively

The behavior of weaned mice at PD 25 in the elevated plus-maze, which was used to evaluate anxiety-like behavior, showed that the number of entries (Fig. 4 a, b) and the time spent (Fig. 4c, d) to explore the open arm in both males and females were significantly increased compared with that of the control group. Additionally, the number of entries to the middle arm was significantly (*p* < 0.001) increased in males (Fig. 4a) exposed to the low concentration (20 g/L), whereas no change was observed in the high-concentration group (50 g/L) compared with that of the control group. Females (Fig. 4b) in the two GTE groups exhibited significantly (*p* < 0.01) more entries relative to that of the control group. The mean number of entries to the closed arm was significantly lower (*p* < 0.001) for males (Fig. 4a) exposed to the two different concentrations of GTE (20 and 50 g/L) compared with that of the control group. For females (Fig. 4b), there was a significant (*p* < 0.01) decline in the number of entries to the closed arm for the low-concentration, without no effect observed for females in the high-concentration group. The time spent exploring the closed arm in both males and females was significantly lower (p < 0.01) compared with that of the control group (Fig. 4c, d). Similarly, the time spent exploring the middle arm only in males (Fig. 4c) was significantly lower (*p* < 0.01) compared with that of the control group. Conversely, the time spent exploring the middle arm in females (Fig. 4d) was significantly (*p* < 0.01) greater in the low-concentration group, with no effects observed for the high-concentration group.

### GTE improved learning and memory of the developing offspring

**Fig. 5:**
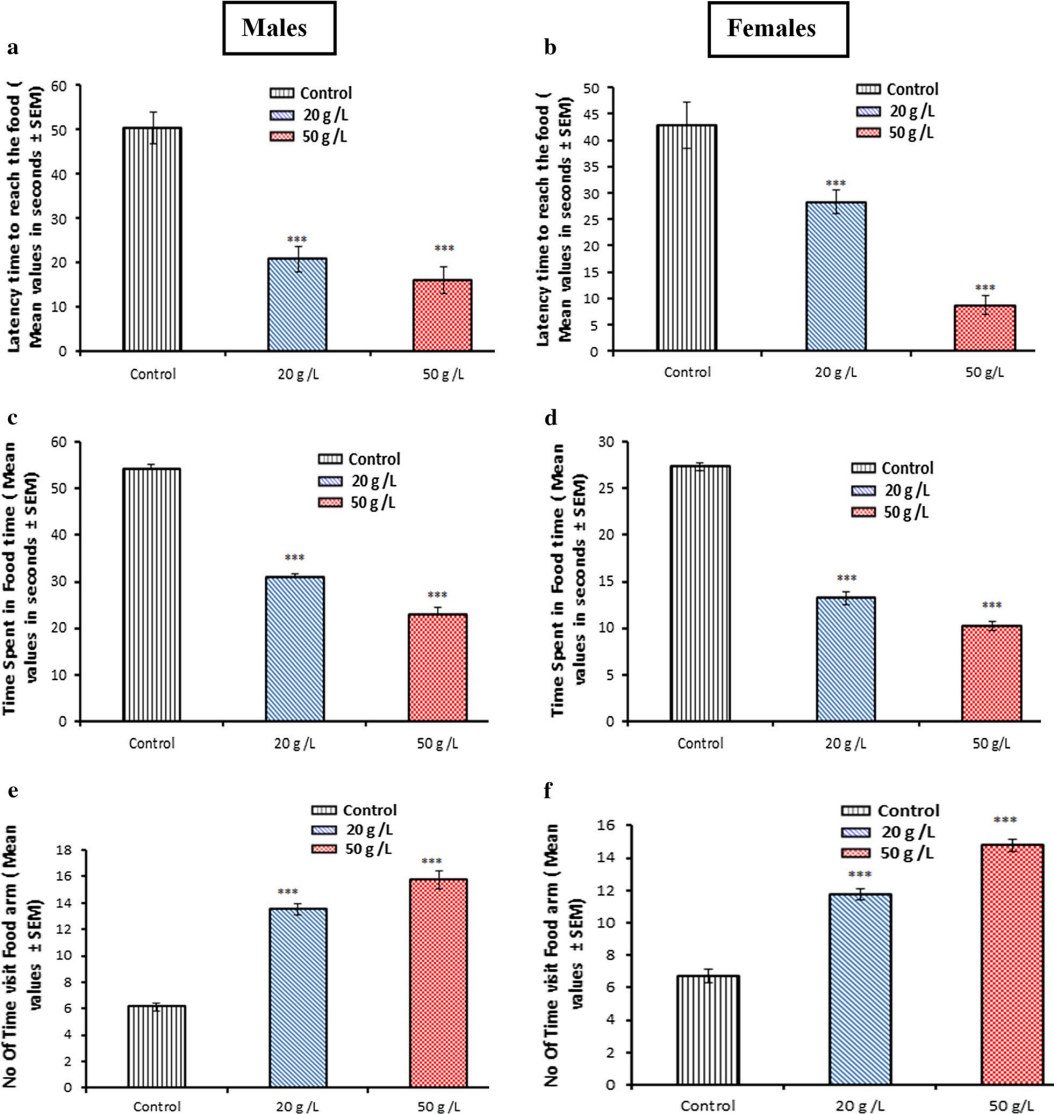
Animal’s behavior in T-maze (learning and memory tests). Latency time to reach the food (**a**, **b**), time spent in food arm (**c**, **d**), number of entries into food arm (**e**, **f**) at postnatal day 30 for mice offspring exposed perinatally to aqueous extract of green tea. Data are presented as the mean ± SEM (*n* = 8). Differences were considered statistically significant at ***p < 0.001

**Fig. 6:**
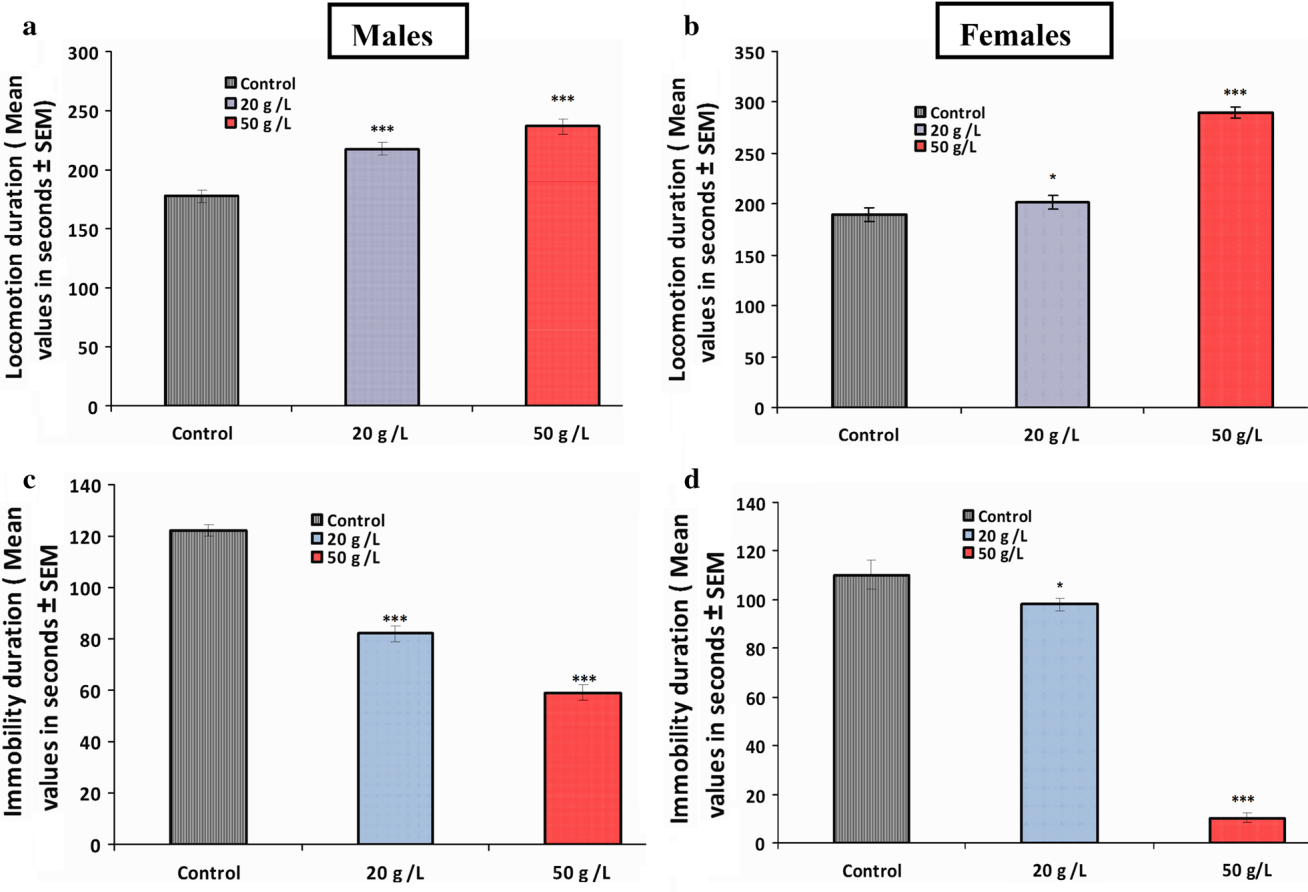
Effects of perinatal GTE exposure on locomotion duration (**a**, **b**) and immobility duration (**c**, **d**) of mice offspring treated perinatally with GTE. Data are presented as the mean ± SEM (*n* = 8). Differences were considered statistically significant at *p < 0.05 and ***p < 0.001

In this study, the elevated T-maze was used to assess learning and memory features in control and experimental mice. The behavior of the animals in the T-maze revealed that latency time to reach the food (Fig. 5a, b) and time spent in the food arm (Fig. 5c, d) were significantly (*p* < 0.001) lower in both males and females of the 20 and 50 g/L groups compared with that of the control group. However, a significant increase (*p* < 0.001) was recorded in number of entries to the food arm (Fig. 5e, f) in both sexes of the two groups (20 and 50 g/L) in comparison with the control group. The number of entries to the empty arm differed between males and females. Analysis of the total duration of locomotion showed a significantly increased response of both males (Fig. 6a) and females (Fig. 6b) in the two experimental groups (20 and 50 g/L) compared with that of the control group. Conversely, a significant decrease was recorded in the total duration of immobility of both males (Fig. 6c) and females (Fig. 6d) of the tested groups when compared with that of the controls.

### Hematological profiles of mice offspring exposed to perinatal GTE

**Fig. 7:**
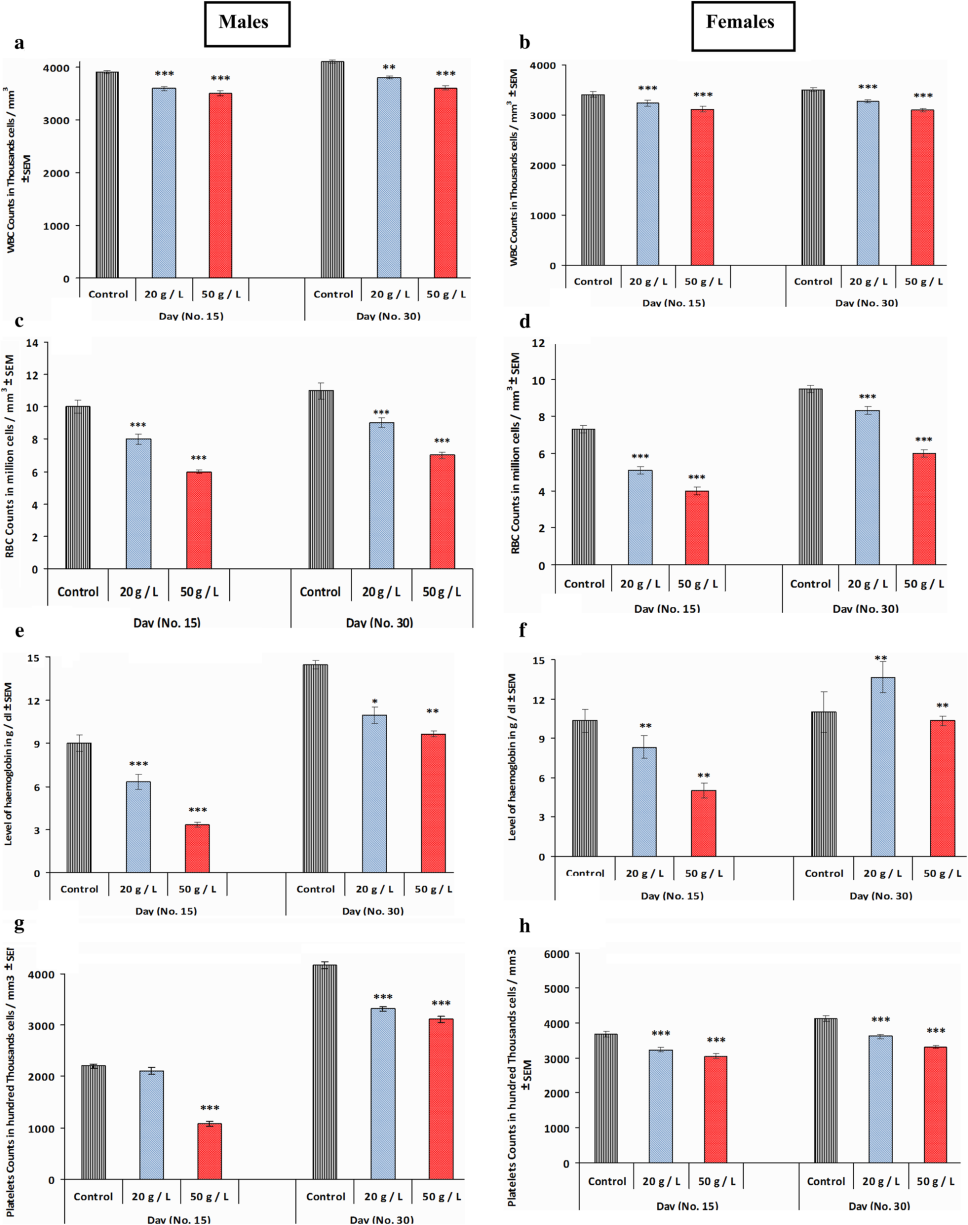
Changes in blood parameters such as WBCs in male offspring (**a**), WBCs in female offspring (**b**), RBCs in male offspring (**c**), RBCs in female offspring (**d**), hemoglobin in male offspring (**e**), hemoglobin in female offspring (**f**), platelets counts in male offspring (**g**) and platelets counts in female offspring (**h**) at PD15 and PD30 following perinatal exposure to GTE. Results are expressed as the mean ± SEM. Differences were considered statistically ignificant at *p < 0.05, **p < 0.01 and ***p < 0.001

Blood profiles were obtained at PD 15 and PD 30. As shown in Fig. 7, except for platelet count of males at PD 15 (Fig. 7g), all hematological parameters (WBCs, RBCs, hemoglobin, and platelets) were significantly decreased by perinatal exposure to GTE in both male and female offspring when compared with those of the control group. There were minor differences between the two groups exposed to the different GT concentrations (20 and 50 g/L).

### Effects of perinatal exposure to GTE on plasma glucose and lipid profile of developing offspring at PD 15 and PD 30

**Fig. 8:**
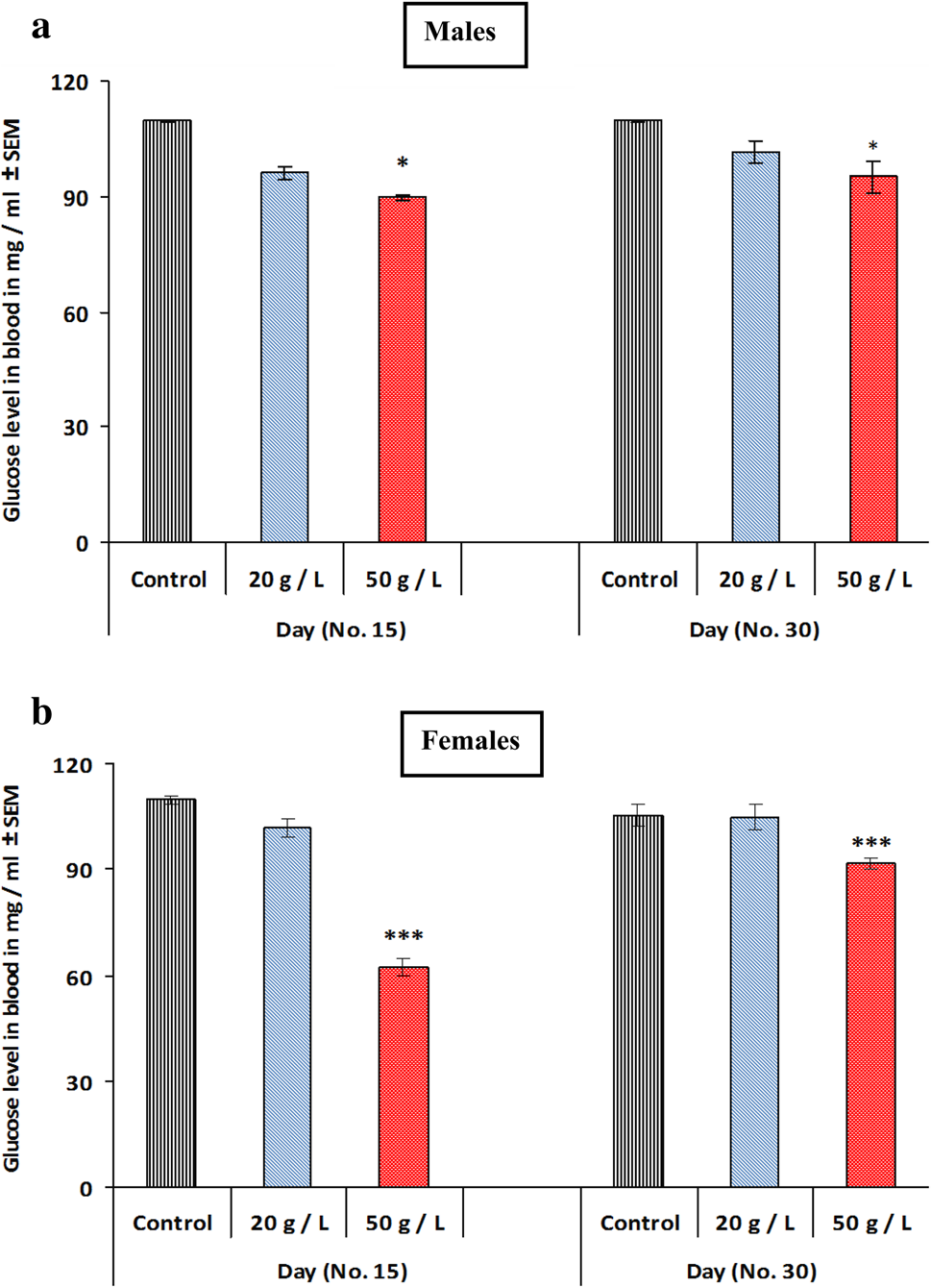
Effects of perinatal exposure to GTE on plasma glucose level (mg/mL) for male offspring (**a**) and female offspring (**b**) at PD15 and PD30. Results are presented as means ± SEM. Statistical significance is indicated by *p < 0.05 and ***p < 0.001

**Fig. 9:**
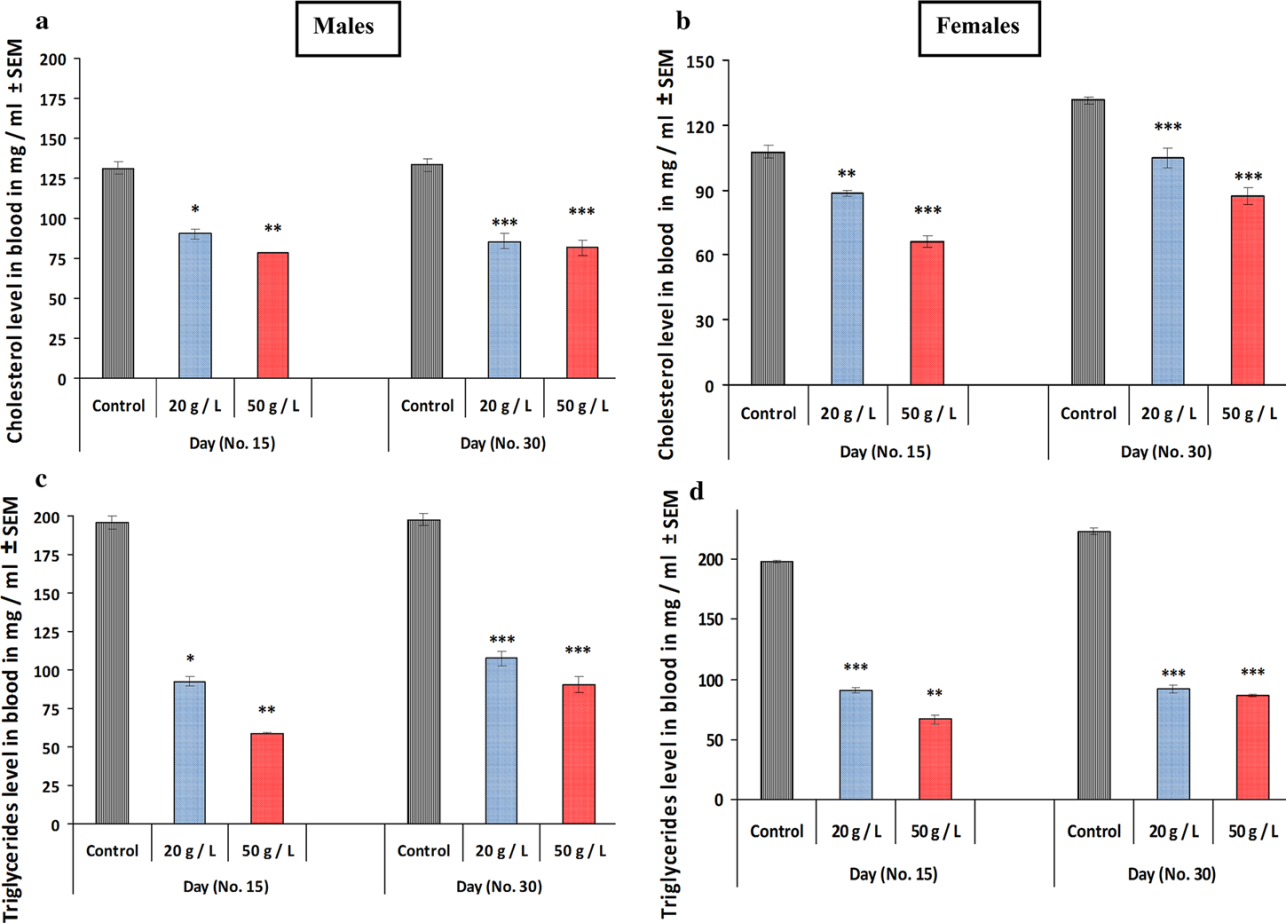
The levels of plasma cholesterol (**a**, **b**) and triglycerides (**c**, **d**) were determined in mice offspring at PD 15 and PD 30 born to dams exposed to perinatal GTE. Results are expressed as the mean ± SEM. Differences were considered statistically significant at *p < 0.05, **p < 0.01, and ***p < 0.001

**Fig. 10:**
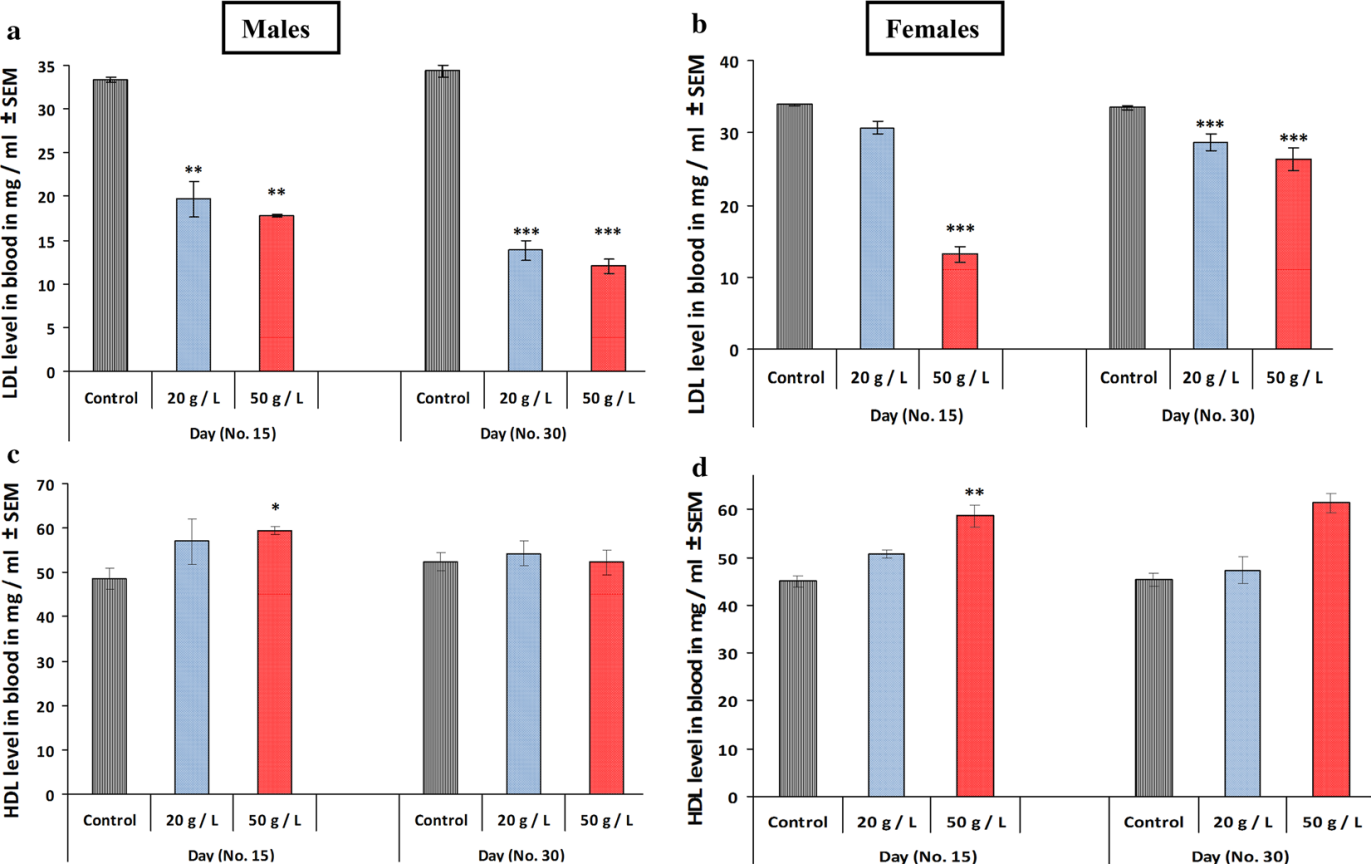
The levels of plasma LDL (**a**, **b**) and HDL (**c**, **d**) were measured in mice offspring at PD 15 and PD 30 born to dams exposed to perinatal GTE. Results are expressed as the mean ± SEM. Differences were considered statistically significant at *p < 0.05, **p < 0.01 and ***p < 0.001

There was a significant decrease (*p* < 0.05, *p* < 0.001, respectively) in plasma glucose levels in mice offspring in the high-concentration group (50 g/L) for GTE when compared to that of the control group at ages PD 15 and PD 30. Similar effects were observed in both male and female offspring (Fig. 8a, b). However, the low-concentration group (20 g/L) exhibited no change for males and females relative to those of the control. Lipid profiles were monitored in the three groups at PD 15 and PD 30 of age. The levels of cholesterol (Fig. 9a, b) and triglycerides (Fig. 9c, d) were significantly lower in the plasma of offspring exposed to both concentrations of GTE (20 and 50 g/L) compared with that of the control group. The decrease was recorded in both male and female offspring and minor differences existed between the two groups. Similarly, perinatal exposure to the two concentrations (20 and 50 g/L) of GTE significantly decreased (*p* < 0.01, *p* < 0.001, respectively) the levels of LDL (Fig. 10a, b) in the plasma of both male and female offspring compared with that of the control group at PD 15 and PD 30; however, its effect in females at PD 15 was non-significant for the low-concentration group (20 g/L) for GTE (Fig. 10b). Conversely, perinatal exposure to the two concentrations (20 and 50 g/L) of GTE resulted in the non-significant effects on the levels of HDL of both male and female mice offspring at PD 30 of age. However, a significant elevation (*p* < 0.05, *p* < 0.01) was recorded in mice offspring exposed the high concentration (50 g/L) of GTE when compared to that of the control group at age PD 15 (Fig. 10c, d).

## Discussion

Behavioral studies on GT are very limited, especially during the embryonic period. The goal of the present study was to explore the impact of perinatal exposure to GTE on the behavior of the developing offspring. In the current experiment on mice, we showed that perinatal exposure to GTE at two concentrations, 20 and 50 g/L, significantly lowered body weight of the developing offspring. Similar observations have been reported previously [28]. Furthermore, offspring born to dams exposed to GTE during gestation and lactation experienced the appearance of body hair and opening of eyes earlier. This might have occurred because GT components crossed via the placenta in utero or they were transmitted in milk during lactation.

In the present study, perinatal exposure to GTE also affected neuromotor reflexes in the weaning pups. GTE exposure resulted in faster responses and had a significant stimulatory effect on the righting reflex, rotating reflex, and cliff avoidance activity. These findings are in accordance with a study [29], which reported the beneficial effects and the positive roles of GTE on the autonomic nervous system based on its ability to penetrate the blood brain barrier and affect brain maturation and development [30]. On the other hand, it has also been shown that maternal caffeine intake, which is one of the components of GT, alters neuromotor development through interfering with cholinergic neurotransmission during brain development [31].

Results from the present study showed an increase in locomotor activity of male and female offspring born to mice exposed to GT during pregnancy and lactation. These results are in accordance with Michna et al. [13], who attributed this to the role of GT in the oxidation of fats, thereby decreasing fat stores and stimulating the central nervous system by caffeine. The elevated Plus-Maze is a paradigm employed to monitor fear and anxiety in mice. Perinatal exposure to GTE produced a significant increase in time spent and number of open arm entries, as well as a significant decrease in time spent and number of closed arm entries in both male and female offspring. This suggested that fear and anxiety decreased and GTE possesses anxiolytic properties. This was supported by the study of Vignes et al. [16] in which it was reported that EGCG had anxiolytic activity. Furthermore, another possible explanation for our findings may be explained by research conducted by Heese et al. [32] in which they documented that l-theanine is a constituent of GT, and when combined with midazolam had anxiolytic effects that might be caused by the alteration of serotonin and/or dopamine metabolism [33].

It was interesting to discover that perinatal exposure to GTE significantly improved learning and memory in male and female offspring, as shown by a decrease in latency time to reach food and time spent in a food arm. Our results are in line with observations of Kaur et al. [34] who attributed this enhancement in learning and memory to the polyphenolic antioxidant constituents of GTE. Further, previous reports have shown that EGCG, the main constituent of GT, produced a marked increase in protein kinase C in the hippocampus, which is the learning site of the brain [35].

Previously, liquid chromatography was used for the detection of the total phenolic and flavonoids constituents in 100 mg GTE [18]. The data confirmed that GTE comprises of total phenolic (35.6 mg) and flavonoids (12.4 mg) constituents per 100 mg of GTE, respectively. The major active biological compounds of GTE were catechein content (31.8 mg/g), pyrogallol (21.8 mg/g), naringenin (11.9 mg/g), catechol (10.9 mg/g), epicatechein (10.5 mg/g), and small amounts of other phenolic compounds. The antioxidant activities and positive effect of green tea could be linked its constituents such as l-theanine and caffeine which have clear beneficial effects on sustained attention, memory, and suppression of distraction. Moreover, l-theanine was found to lead to relaxation by reducing caffeine induced arousal [36]. Additionally, green tea polyphenols produced antidepressent like effect in adult mice [17]. Green tea extract and catechin also ameliorated chronic fatigue-induced oxidative stress [37].

Our results showed that perinatal exposure to GTE significantly decreased all hematological parameters (RBCs, WBCs, Hb, and platelets) in both male and female offspring. The decrease in RBCs and Hb might have been caused by the effects of tea polyphenols on iron bioavailability and absorption [38], whereas the decrease in platelets might have been caused by GT catechins, which decrease platelet stickiness and aggregation [39]. The decrease in WBCs might have been associated with the decrease in RBCs and platelets.

The high-concentration group for GTE exhibited hypoglycemic effects and a significant decrease in blood glucose levels in male and female offspring. EGCG has been found to control blood sugar through inhibition of its absorption in the intestine by the sodium-dependent glucose transporter SGLT1 [40]. GTE also significantly lowered blood cholesterol and triglyceride levels, which might have been caused by its inhibitory effect on the intestinal absorption of lipids [41]. Similar findings have been reported previously [42]. Herein, GTE significantly lowered blood LDL, without altering HDL, concentrations. However, a significant increase in HDL was recorded in case of exposure to GTE at high concentration. Similar observations were made by Zheng et al. [43] in a study from which it was reported that GT catechins affect lipid metabolism.

## Conclusion

Perinatal exposure to GTE had anxiolytic properties and positive effects on locomotor activity, sensory motor reflexes, as well as learning and memory of the subsequent offspring. The positive effects of GTE could be related to the antioxidant properties of its constituents.

## Abbreviations

GT: green tea
GTE: green tea extract
PD: postnatal Day
EGCG: epigallocatechin-gallate
RBCs: red blood cells
WBCs: white blood cells
Hb: hemoglobin
HDL: high density lipoproteins
LDL: low density lipoproteins
